# The Ubiquitin-Proteasome Pathway in Huntington's Disease

**DOI:** 10.1100/tsw.2008.60

**Published:** 2008-04-20

**Authors:** Siddhartha Mitra, Steven Finkbeiner

**Affiliations:** ^1^ Gladstone Institute of Neurological Disease, 1650 Owens St., San Francisco, CA 94158, USA; ^2^ Medical Scientist Training Program, University of California, 533 Parnassus Ave, San Francisco, CA 94143, USA; ^3^ Departments of Neurology and Physiology, University of California, 533 Parnassus Ave., San Francisco, CA 94143, USA

**Keywords:** neurodegeneration, polyglutamine, autophagy, protein misfolding

## Abstract

The accumulation of mutant protein is a common feature of neurodegenerative disease. In Huntington's disease, a polyglutamine expansion in the huntingtin protein triggers neuronal toxicity. Accompanying neuronal death, mutant huntingtin aggregates in large macromolecular structures called inclusion bodies. The function of the machinery for intracellular protein degradation is linked to huntingtin toxicity and components of this machinery colocalize with inclusion bodies. An increasing body of evidence implicates the ubiquitin-proteasome pathway in the failure of cells to degrade mutant huntingtin. A number of potential mechanisms that link compromised ubiquitin-proteasome pathway function and neurodegeneration have been proposed and may offer opportunities for therapeutic intervention.

## THE BURDEN OF NEURODEGENERATIVE DISEASE

Neurodegenerative disease is the sixth leading killer in the U.S. Alzheimer's disease, Parkinson's disease, amyotrophic lateral sclerosis, and Huntington's disease (HD) all affect different regions of the brain and have heterogeneous clinical presentations. However, each increases in incidence with age, affects the nervous system, and inexorably leads to death. Each is also characterized by accumulation of misfolded mutant protein. Among them, only HD is predominantly inherited, with a single gene at the root of the disease. As in the case of other neurodegenerative diseases, there is no cure for HD, nor is there any treatment that slows progression.

## HUNTINGTON'S DISEASE

HD is estimated to affect five to seven in 100,000 people, although up to 90% fewer are affected in Asian and African populations[1]. While HD most often presents in the fourth or fifth decade of life, the onset of disease has been documented from ages of 1 through 80 years, with changes in personality, motor control, and cognition[2,3]. At the time of clinical diagnosis, most patients demonstrate chorea or dystonia and rigidity, a lack of coordination, saccadic eye movements, and an inability to voluntarily maintain muscle tone[1].

HD belongs to a family of nine diseases caused by an expansion of a stretch of CAG repeats in the coding region of a gene. The effect of the CAG repeat expansion is dependent on the gene context of the repeat; the brain regions first affected by each disease vary as do the threshold number of repeats needed to cause disease. With the exception of X-linked spinobulbar muscular atrophy, which is caused by a CAG expansion in the coding region of the androgen receptor[4], all are autosomal dominant in inheritance. Spinocerebellar ataxias (SCA) 1, 2, 3, 6, 7, and 17 are caused by CAG repeat expansions in the ataxin 1, 2, 3, 6, 7, and TBP genes and affect primarily the cerebellum and brainstem; dentatorubral-pallidoluysian atrophy is caused by a CAG repeat expansion in the atrophin 1 gene and affects the cerebellum as well as the cortex and midbrain[5,6,7,8].

The gene responsible for HD was identified in 1993 and codes for a widely expressed 348-kDa protein with a polymorphic CAG trinucleotide repeat[9]. The CAG repeats translate into a homomeric polyglutamine (polyQ) stretch in the amino terminus of the huntingtin (htt) protein. HD develops when the polyQ stretch exceeds a threshold of 36 glutamines. The length of the polyQ stretch is inversely proportional to the age of onset of the disease; polyQ stretch length may explain more than 70% of the variation in the age of disease onset[10,11,12,13] and can be used in a parametric model to predict disease incidence accurately[14].

## SELECTIVE NEURONAL DEATH

As HD progresses, mutant htt leads to the death of specific neuronal subpopulations. Medium spiny neurons in the striatum that express enkephalin and γ-aminobutyric acid are particularly susceptible to mutant htt[15]. The medial paraventricular region and tail of the caudate, along with the dorsal putamen, are the first brain regions to show significant atrophy[16]. Neuronal death spreads to the cortex, white matter, and thalamus later in the disease[17,18]. Although htt is widely expressed in the brain and other tissues[19,20], the level of expression varies by brain region and cell type. This variation may explain part of the specificity of cell death in HD[21].

## GAIN OF TOXIC FUNCTION

The genetics of HD and other CAG repeat disorders strongly support the hypothesis that polyQ expansion leads to a toxic activity that is not present for shorter polyQ stretches. HD is inherited in an autosomal-dominant pattern with almost complete penetrance when the gene contains more than 40 CAG repeats[14]. In mice, the deletion of *Hdh*, the mouse *HTT* homolog, results in embryonic lethality[22,23]. While supporting the possibility that the normal function of htt is required during brain development, this loss-of-function phenotype is quite distinct from that seen in HD[24]; furthermore, mutant htt is sufficient to rescue the embryonic-lethal loss-of-function phenotype, arguing that mutant htt retains significant activity and that mutant htt–induced pathology is distinct from a loss-of-function phenotype[25]. Cell-specific inactivation of *Hdh* in the brain and testis of mice results in age-related neurodegeneration, but neither the pathology nor the phenotype resembles HD models[26]. In contrast, a fragment of mutant htt composed of only the first exon is sufficient to cause a progressive neurodegenerative phenotype resembling HD[27]. Similarly, a long polyQ expansion in the context of full-length htt in mice results in behavioral changes and neuropathology characteristic of HD[28,29].

## HTT INCLUSION BODIES ARE A HALLMARK OF HD

Accompanying the gain of toxic function, full-length mutant htt is cleaved into amino-terminal fragments that are deposited in large, microscopically visible, inclusion bodies (IBs) in the nucleus, cytoplasm, and processes of neurons[30,31]. IBs form in populations of neurons in humans and mice that undergo death and dysfunction in the course of disease[30,31,32]. The formation of large, microscopically visible, intracellular aggregates is not unique to HD; the pathology of other diseases caused by polyQ expansion is also marked by the formation of IBs by each of the respective causative proteins.

IB formation in polyQ diseases is mediated, at least in part, by the polyQ expansion itself[33], but other proteins are also present in these structures. Many components of the intracellular machinery responsible for handling and degrading misfolded protein, including chaperones, ubiquitin, proteasome subunits, and autophagic machinery, can be found in mutant htt IBs[30,31,34,35,36]. The colocalization of these proteins was one of the first clues that the regulation of intracellular mutant htt levels is central to HD pathogenesis.

## HD PATHOLOGY IS REVERSIBLE

Even stronger evidence for the role of intracellular levels of mutant htt in HD pathogenesis comes from studies of an inducible mouse model of HD[37,38]. This model demonstrates neuropathology and behavioral changes characteristic of HD when the mutant htt transgene is expressed. If the transgene is shut off rather than remaining expressed after an initial period of induction, the decrease in burden of mutant htt leads to a decrease in the number of IBs, a decrease in brain atrophy, and an improvement in the motor phenotype of the mice. This reversal was dependent on the function of one major pathway for intracellular protein degradation, the ubiquitin-proteasome pathway (UPP). These results offered the possibility that UPP function might suppress mutant htt toxicity and that intracellular protein degradation may be impaired in the course of disease. Although these results also suggested that reducing IB formation would improve mutant htt toxicity, the relationship between IBs and HD pathogenesis has proved to be more complex (Fig. 1).

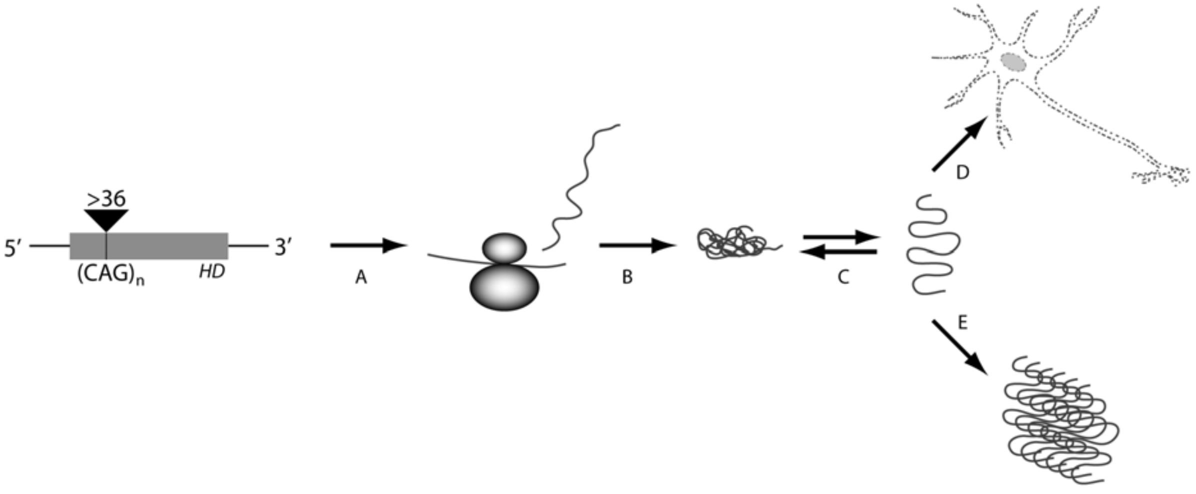

**FIGURE 1.** A model of HD pathogenesis. HD is initiated by the expansion of a CAG stretch in the HD gene that codes for a polyQ stretch in the amino terminus of the htt protein (A). Chaperones, such as Hsp40 and Hsp70, regulate the folding of nascent polypeptides (B) and regulate the folding of mutant htt (C). The amount of intracellular mutant htt, likely of a particular conformation, predicts both neuronal toxicity (D) and IB formation (E). Since cells with IBs survive better than those without, it is likely that IB formation is a protective cellular response and unrelated to the primary mechanism by which mutant htt is toxic.

## MUTANT HTT, IBs, AND TOXICITY

Although many manipulations of yeast, mammalian cell culture, and transgenic mouse models show a correlation between IB formation and toxicity, an accumulating body of evidence demonstrates that IB formation is not necessary for htt toxicity and may instead be a protective cellular response. IB formation and toxicity both increase as a result of increasing expression of mutant htt protein, heat shock of cells, or by inhibiting chaperone function[39,40,41], while they decrease from decreasing mutant htt expression, overexpressing chaperones, activation of the IGF-1/Akt pathway, or overexpression of intracellular protein degradation machinery[42,43,44,45]. The presence of mutant htt IBs, however, can be dissociated from mutant htt toxicity. Low levels of transgene expression in mice expressing a yeast artificial chromosome encoding full-length mutant htt with 72 glutamines is sufficient for neuropathology with no IBs present[46]. In a primary neuronal culture system, cells that form mutant htt IBs survive better than those that do not. Instead, the amount of mutant htt on an individual cell basis predicts how long the cells will survive[47].

To better understand the mechanism by which mutant htt levels may mediate HD pathogenesis, a closer examination of intracellular protein turnover is warranted. The literature supporting the role of autophagy in HD and neurodegeneration was recently reviewed[48,49]. We will focus on the role of the UPP in HD pathogenesis here.

## POLYUBIQUITIN CHAINS MARK PROTEINS FOR DEGRADATION

The UPP is the major pathway for regulated intracellular protein degradation (Fig. 2). Proteins are tagged for degradation by the conjugation of a minimum of four ubiquitins in a polyubiquitin chain[50]. Ubiquitin, a 76-amino-acid polypeptide, is first activated and then conjugated by sequential ATP-dependent thioester linkages to cysteine residues on E1 ubiquitin–activating enzymes and E2 ubiquitin–conjugating enzymes[51]. With the assistance of an E3 ubiquitin ligase, ubiquitin forms an isopeptide bond with the side-chain amino group of lysine residues on substrate proteins[52,53,54]. Several internal lysine residues within ubiquitin can then be used as sites for additional ubiquitin conjugation: chain elongation through K48 is the canonical signal for protein degradation[50]. For some substrates, an E4 ubiquitin chain elongation factor may enhance the efficiency of polyubiquitin chain formation[55,56].

Ubiquitin conjugation is in competition with other modifications of substrate lysines and in opposition to the activity of deubiquitinating enzymes. Modifications, such as the addition of the small ubiquitin-like modifier-1 (SUMO-1), can shunt proteins away from the UPP[57]. Deubiquitinating enzymes, which are predominantly cysteine proteases, play critical roles in processing ubiquitin precursors and removing ubiquitins from protein substrates[58,59]. While an increase in deubiquitination activity and the removal of ubiquitin from substrates early in the targeting process may delay or prevent protein degradation, a decrease in deubiquitinating activity may also decrease protein degradation by reducing the pool of free ubiquitin intracellularly[60].

## PROTEASOMES DEGRADE POLYUBIQUITINATED SUBSTRATES

Once proteins are polyubiquitinated, they are delivered to the proteasome for degradation. The 26S proteasome is a multisubunit complex composed of 19S regulatory and 20S core particles[61]. The 20S core is a cylindrical structure composed of four stacked heteroheptameric ring complexes. Subunits with trypsin-like, chymotrypsin-like, and peptidylglutamyl peptide hydrolase activities are contained in the two inner rings[62,63]. These activities are derepressed when the 20S core is bound by ATP-hydrolyzing subunits of the 19S regulatory particle[64,65]. The 19S particle binds to polyubiquitin chains on degradation substrates, releases ubiquitins, and unfolds and translocates substrates into the central chamber of the 20S core[61,62]. In some cases, the 11S regulatory particle, alternatively designated REG

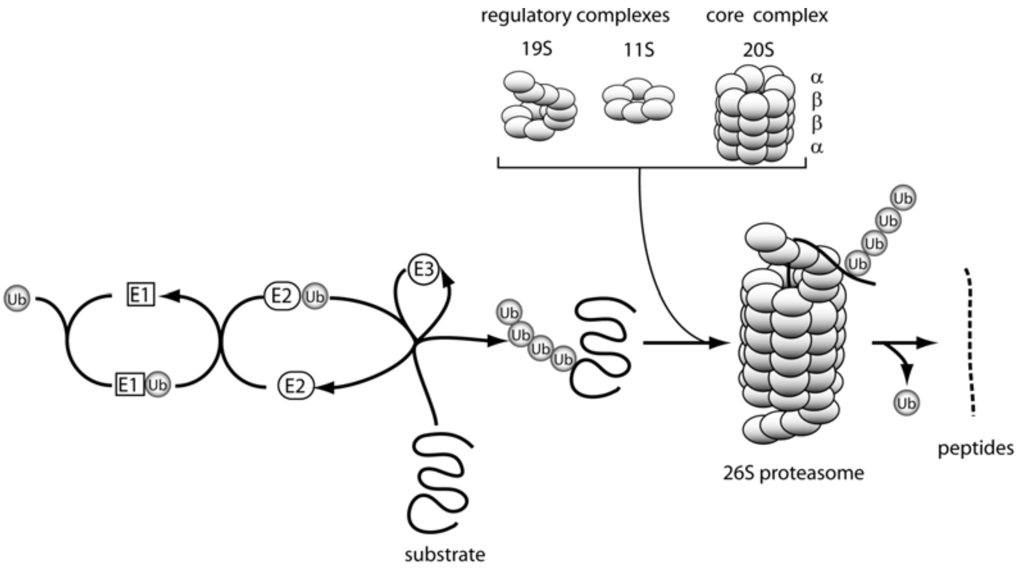

**FIGURE 2.** The UPP. Ubiquitin (Ub) is activated by conjugation to an E1 ubiquitin–activating enzyme and transferred to an E2 ubiquitin–conjugating enzyme, which in concert with an E3 ubiquitin ligase, transfers ubiquitin to a target lysine on a substrate protein. Additional ubiquitins can be added to the already conjugated ubiquitin, resulting in a polyubiquitin chain. Once a polyubiquitin chain formed by sequential lysine 48 linkages is conjugated to a target, the substrate is degraded by the 26S proteasome. The 19S regulatory cap binds the polyubiquitin chain, removes the ubiquitins, unfolds the substrate, and activates the 20S proteolytic core in an ATP-dependent manner. The 11S regulatory complex performs an analogous role to the 19S complex, but in a ubiquitin and ATP-independent fashion. The 20S core is organized in four heptameric rings, with tryptic, chymotryptic, and peptidylglutamyl proteolytic activities contained in the inner β rings. Peptides released by the proteasome may be further degraded by intracellular peptidases.

or PA28, substitutes for the 19S complex and leads to changes in substrate preference and proteolytic activity[66,67,68,69,70]. In contrast to activation by the 19S particle, PA28 activation of the 20S core is ATP independent, and PA28 mediates ubiquitin-independent protein degradation. This activity may be particularly important for the degradation of small peptide fragments[71,72].

## TARGETING MUTANT HTT TO THE UPP

Ubiquitin and mutant htt colocalize in brain tissue from HD patients[30,73], HD mouse models[31], and HD cellular models[74,75]. Biochemical analysis demonstrates that ubiquitin is present in mutant htt IBs[34,76] and that mutant htt is itself ubiquitinated[75,77,78]. Ubiquitination of mutant htt is in competition with modification by SUMO-1, which reduces IB formation and exacerbates mutant htt toxicity[78]. Polyubiquitinated mutant htt is targeted to the proteasome for degradation (Fig. 3). After proteasome inhibition, ubiquitinated species of mutant htt accumulate, and the amount of aggregated mutant htt increases[75,77,79,80,81].

The machinery responsible for ubiquitinating specific substrates in mammalian systems has been difficult to identify, and the case of mutant htt is no different. There are more than 50 E2-conjugating enzymes and over 500 E3 ligases in the human genome[82]. Much of the substrate specificity for ubiquitination has been ascribed to E3 ligases, although the determinants of specificity are poorly understood. The challenge of identifying the ligase that ubiquitinates a particular protein substrate is akin to that of identifying a kinase responsible for phosphorylating a particular target — functional compensation muddles the interpretation of genetic approaches, and functional redundancy leads to difficulties in interpreting experiments from reconstituted systems.

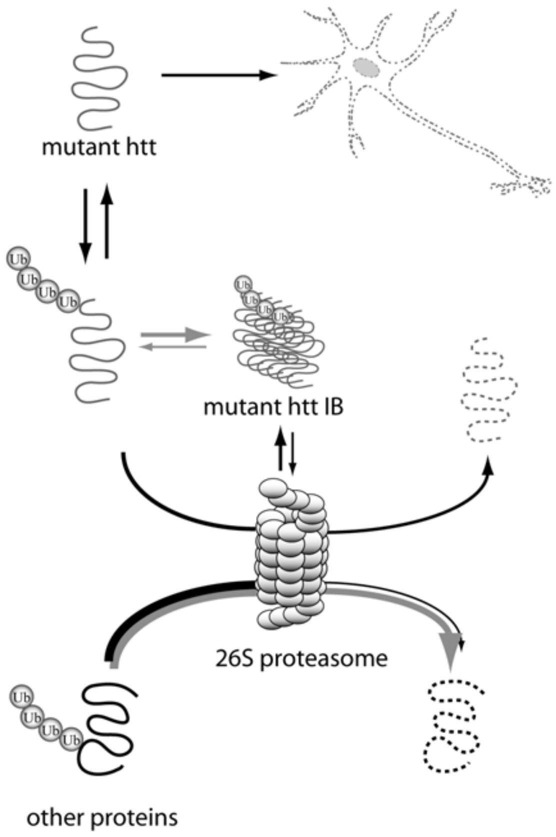

**FIGURE 3.** Mutant htt and the UPP. The ubiquitination and degradation of mutant htt by the proteasome decreases neuronal dysfunction and death in cellular and animal models of HD. However, mutant htt impairs UPP function, with an accompanying intracellular accumulation of ubiquitinated proteins. The impairment of UPP function may lead to toxicity by an increase in the amount of intracellular mutant htt, an increase in the levels of other proteins targeted for degradation, or a combination thereof. Although cells with IBs show high levels of UPP impairment, and ubiquitin and some proteasome subunits localize to IBs, UPP impairment can occur independently of IB formation. Interestingly, while proteasome subunits are recruited to actively forming IBs, preformed IBs *in vitro* do not have this activity. Although it is hard to reconcile high levels of UPP impairment with improved survival in cells with mutant htt IBs, one possibility is that IB formation may be one mechanism by which the cell may improve the degradation of other cellular proteins (gray lines with htt IB formation and black lines with htt flux through the UPP).

Although determining what machinery is physiologically responsible for the degradation of mutant htt has not yet been possible, UPP components that affect the ubiquitination, degradation, and toxicity of mutant htt have been identified. hE2-25K, a ubiquitin-conjugating enzyme, interacts with the first 540 amino acids of mutant htt[83], increases mutant htt IB formation, and decreases toxicity in cell culture[84]. A dominant-negative form of another E2, cdc34, decreases the number of IBs in cell culture and potentiates neuronal death due to mutant htt[74]. When overexpressed, the E3 ubiquitin ligase Hrd1 is found in complexes with htt, increases the amount of ubiquitinated mutant htt, and decreases the total amount of mutant htt in cell culture. Hrd1 overexpression also decreases the number of mutant htt IBs and mutant htt toxicity[85]. The E3 ubiquitin ligase parkin colocalizes with mutant htt in the brains of HD

patients and HD mouse models. Parkin reduces the levels of polyQ-containing proteins through its ubiquitin ligase activity[45]. The E4 protein CHIP colocalizes with mutant htt and, when overexpressed, increases mutant htt ubiquitination, decreases IB formation, and improves survival in a cellular model of HD[86]. Loss of Ubp13, the yeast homolog of the human deubiquitinating enzyme Usp12, enhances mutant htt toxicity in yeast[87].

While these results demonstrate that the UPP can degrade mutant htt and modulate htt toxicity, the roles of hE2-25K, cdc34, Hrd1, parkin, CHIP, and Usp12 in HD pathogenesis remain unclear. These genes may modulate mutant htt IB formation and toxicity by a direct effect on mutant htt ubiquitination, an effect on the ubiquitination of other substrates, or an effect on the activity or intracellular localization of interaction partners. No matter the mechanism, the result of these effects is consistent with the ubiquitination of mutant htt enhancing mutant htt degradation and reducing mutant htt toxicity (Fig. 3).

## MUTANT HTT IMPAIRS UPP FUNCTION

The hypothesis that the polyQ expansion in mutant htt impairs UPP function originated from the observation that components of the UPP are stably associated with mutant htt in IBs. While IBs in other neurodegenerative diseases, such as SCA1 or ALS, have a fast component of protein exchange between IBs and the cellular milieu[88,89,90], IBs with mutant htt appear to be largely static in cell culture. Eukaryotic proteasomes degrade polyQ sequences poorly in reconstituted systems[91,92] and may release polyQ peptides for further degradation by cytosolic aminopeptidases[93]. These results have led to the hypothesis that polyQ peptides may be transiently retained in the proteolytic core and impair proteasome activity, but it is equally possible that the propensity for mutant htt to aggregate may lead to difficulties with targeting mutant htt to the proteasome or for the proteasome regulatory complex to unfold mutant htt and present it to the core catalytic subunits for proteolysis.

Mutant htt impairs the ability of the UPP to degrade other intracellular protein substrates, but this effect is not likely due to the recruitment of UPP components into static IBs (Fig. 3)[94,95,96]. Although UPP machinery colocalizes with htt IBs, the majority of proteasome subunits are not recruited to these structures in cell culture models[95]. While the accumulation of proteasome substrates is pronounced in cells with mutant htt IBs[94], UPP impairment occurs without IB formation[95].

By impairing UPP function, mutant htt establishes an unstable equilibrium that may underlie HD pathogenesis. As mutant htt causes UPP substrates to accumulate, mutant htt levels increase and worsen UPP impairment. In response to increasing levels of UPP impairment, the cell may deliver substrates to the autophagic pathway of intracellular protein degradation through mediators, such as HDAC6[97], and attempt to reestablish cellular homeostasis[48].

## MEDIATORS OF UPP IMPAIRMENT IN HD

While mutant htt clearly impairs UPP function, the role of this impairment in HD has yet to be elucidated. UPP impairment may have potentially subtle changes on a large number of proteins, complicating the identification of effectors of UPP dysfunction. Mutant htt and other proteins prone to misfold may establish positive feedback circuits for UPP impairment and cellular dysfunction[98]. The interaction of these substrates may be indirect and coordinated by chaperones. Protein interaction partners may also modulate reciprocal, metastable, conformational changes in protein structure akin to prion conversion through interaction domains, such as polyQ stretches[99]. These conformational changes may promote or inhibit cellular dysfunction and death.

Inhibition of the proteasome in proliferating cells causes cell-cycle arrest and ensuing cell death. If UPP impairment leads to toxicity through an accumulation of toxic protein, potential mediators include Rb, p53, E2F, p21, p27, cyclin D, cyclin E[100], BIK, NOXA, BIM[101], and AIF[102]. The relationships among these substrates, UPP function, and cellular toxicity in postmitotic cells are unclear.

One intriguing potential mediator of UPP impairment is a mutant form of ubiquitin-B (UBB+1). UBB+1 is found in a wide range of diseases, including Alzheimer's disease[103] and polyQ diseases[104], and demonstrates a dose-dependent conversion between UPP substrate and UPP inhibitor[105]. UBB+1 is a particularly attractive candidate for the role of a molecular switch that could induce cell death and prevent the accumulation of a large burden of insoluble, aggregated protein.

UPP function is critical for the maintenance of long-term potentiation (LTP)[106] and for regulating protein synthesis[107] in neurons. A careful analysis relating UPP impairment and LTP or protein synthesis may be fruitful to understand early neuronal dysfunction in the course of HD.

## UPP FUNCTION AND AGING

The dependence of neurodegenerative disease on age may partially be explained by the combination of an increased burden of misfolded protein and a decline in UPP function. As HD mice age, nuclear and cytoplasmic proteasome activities decline in the cortex, striatum, and cerebellum[80]. An increased proportion of cross-linked or oxidized proteasome subunits may accompany the aging process[108]. Intriguingly, the function of an E3 ubiquitin ligase in *C. elegans* regulates longevity[109], offering the possibility that UPP function may affect neurodegenerative disease by changing the rate of aging. Further investigation into the interaction between age and UPP function will help to determine if UPP impairment contributes to the age dependence of HD.

## THERAPEUTIC STRATEGIES AND FUTURE DIRECTIONS

Although the identity of the UPP machinery responsible for degrading mutant htt remains unclear, the UPP likely plays a central role in HD pathogenesis. UPP impairment is well documented in the pathogenesis of neurodegenerative disease. Mutations in the E3 ubiquitin ligases parkin and E6-AP cause Parkinson's disease and Angelman's syndrome, respectively, while mutations in the ubiquitin protease ataxin-3 cause Machado-Joseph's disease[110,111,112,113,114]. In each of these diseases, a decrease in the activity of a particular UPP component leads to the dysfunction and death of particular subsets of neurons.

Improving the function of the UPP may be one approach to prevent the progression of HD and neurodegenerative disease generally. However, strategies to increase UPP function will have a multitude of off-target effects unless the machinery specifically responsible for the turnover of particular mutant proteins is targeted.

Molecular links between UPP impairment and HD pathogenesis have yet to be identified. Some of this machinery must have cell type–specific activity if the neuronal specificity of cell death in HD is explained by UPP impairment. The relative contribution of the UPP and the autophagic pathway in the turnover of mutant htt will also be important to determine. The efficacy of strategies to treat HD that improve UPP function will likely be dependent on the strength of the relationship between UPP impairment and HD pathogenesis.

## ACKNOWLEDGMENTS

We thank members of the Finkbeiner lab, in particular, Gaia Skibinski and Andrey Tsvetskov, for helpful discussions. We thank Sarah Minick for assistance in preparation of the figures, Kelley Nelson for assistance in the preparation with the manuscript, and we thank Gary Howard and Stephen Ordway for editorial assistance. S.M. is supported by the NIH-NIGMS UCSF Medical Scientist Training Program. S.F. is supported by the National Institutes of Neurological Disorders and Stroke (NS039074 and

NS045191), the National Institute of Aging (AG022074), the Taube Family Foundation, and the J. David Gladstone Institutes.
